# Intrathecal administration of colistin, vancomycin and amikacin for central nervous system infections in ICU neurosurgical patients

**DOI:** 10.1186/cc14202

**Published:** 2015-03-16

**Authors:** P Alexandropoulos, S Georgiou, V Chantziara, A Tsimogianni, E Chinou, V Karagiannisa, G Michaloudis

**Affiliations:** 1 Saint Savvas Oncology Hospital, Athens, Greece

## Introduction

Central nervous system (CNS) infections in ICU patients after neurosurgery are a difficult and life-threatening complication demanding immediate action. In many cases intravenous (i.v.) administration of antibiotics is not sufficient; thus, intrathecal (i.t.) administration is required.

## Methods

From January 2013 to November 2014 all cases with CNS infections were recorded. Inclusion criteria were the presence of fever ≥38.5°C, increased inflammatory markers, compatible lumbar puncture (LP) findings (increased number of polymorphonuclear leukocytes, increased protein and low glucose compared with serum levels) and no evidence of other site of infection. All subjects were receiving appropriate i.v. antibiotic treatment based on cultures. Intrathecal administration of 300,000 iu colistin, 25 mg vancomycin and 25 mg amikacin was performed taking under consideration that neurosurgical patients in the ICU have CNS infection attributed to Gram-negative bacteria or/and to *Staphylococcus *species.

## Results

Overall, nine cases with CNS infection were recorded aged from 22 to 74, all males. LP was performed between the second and 17th day (average 8.3 days) and the CSF analysis showed 40 to 6,000 cells - mainly PMNs, protein 161 mg% to 287 mg% and glucose from 3 to 58 mg/dl. They were all colonized with *Acinetobacter baumannii *sensitive only to colistin. CSF cultures were negative for all patients besides one, who grew *A. baumannii*. Of those, seven (77%) were receiving i.v. colistin, eight (88%) carbapenems, and eight (88%) glycopeptides, all in combination with other antibiotics. Median i.t. administration time was 9.1 days. All patients responded to i.v. and i.t. antibiotics but there was one case in which fever relapsed and increased number of cells in subsequent LP was observed which was attributed to colistin, which was withdrawn. All these patients survived, and were discharged to the ward.

## Conclusion

Patients treated with the abovementioned regime showed clinical and biochemical improvement. The above drug combination turned out to be successful in neurosurgical ICU patients with CNS infection.
